# BluePrint breast cancer molecular subtyping recognizes single and dual subtype tumors with implications for therapeutic guidance

**DOI:** 10.1007/s10549-022-06698-x

**Published:** 2022-08-19

**Authors:** Midas M. Kuilman, Architha Ellappalayam, Andrei Barcaru, Josien C. Haan, Rajith Bhaskaran, Diederik Wehkamp, Andrea R. Menicucci, William M. Audeh, Lorenza Mittempergher, Annuska M. Glas

**Affiliations:** 1 Department of Research and Development, Agendia N.V, Radarweg 60, 1043 NT Amsterdam, The Netherlands; 2 Department of Medical Affairs, Agendia Inc, 22 Morgan, Irvine, CA 92618 USA

**Keywords:** Genomic testing, BluePrint, Breast cancer, Single and dual subtypes, Molecular subtypes

## Abstract

**Purpose:**

BluePrint (BP) is an 80-gene molecular subtyping test that classifies early-stage breast cancer (EBC) into Basal, Luminal, and HER2 subtypes. In most cases, breast tumors have one dominant subtype, representative of a single activated pathway. However, some tumors show a statistically equal representation of more than one subtype, referred to as dual subtype. This study aims to identify and examine dual subtype tumors by BP to understand their biology and possible implications for treatment guidance.

**Methods:**

The BP scores of over 15,000 tumor samples from EBC patients were analyzed, and the differences between the highest and the lowest scoring subtypes were calculated. Based upon the distribution of the differences between BP scores, a threshold was determined for each subtype to identify dual versus single subtypes.

**Results:**

Approximately 97% of samples had one single activated BluePrint molecular subtype, whereas ~ 3% of samples were classified as BP dual subtype. The most frequently occurring dual subtypes were the Luminal-Basal-type and Luminal-HER2-type. Luminal-Basal-type displays a distinct biology from the Luminal single type and Basal single type. Burstein’s classification of the single and dual Basal samples showed that the Luminal-Basal-type is mostly classified as ‘luminal androgen receptor’ and ‘mesenchymal’ subtypes, supporting molecular evidence of AR activation in the Luminal-Basal-type tumors. Tumors classified as Luminal-HER2-type resemble features of both Luminal-single-type and HER2-single-type. However, patients with dual Luminal-HER2-type have a lower pathological complete response after receiving HER2-targeted therapies in addition to chemotherapy in comparison with patients with a HER2-single-type.

**Conclusion:**

This study demonstrates that BP identifies tumors with two active functional pathways (dual subtype) with specific transcriptional characteristics and highlights the added value of distinguishing BP dual from single subtypes as evidenced by distinct treatment response rates.

**Supplementary Information:**

The online version contains supplementary material available at 10.1007/s10549-022-06698-x.

## Introduction

Breast cancer (BC) is a heterogenous disease with respect to clinical, histopathological, and molecular features. Based on clinical behavior and genomic characteristics, multiple methods have been utilized to categorize BC into distinct subgroups, be it with clinical subtyping for hormone receptor (HR) protein status or more recently with molecular subtyping based on RNA assays [1–4].

Clinical subtyping relies on well-established immunohistochemistry (IHC) and fluorescence in situ hybridization (FISH) staining that determines estrogen receptor (ER), progesterone receptor (PR), and human epidermal growth factor receptor 2 (HER2) status [5, 6]. The BluePrint (BP) 80-gene subtyping assay was developed to bridge clinical pathology and molecular subtyping, by using IHC-based receptor status and mRNA expression, resulting in a molecular diagnostic array with predictive value [1, 7]. Each of the three subtypes determined by BP (Basal-type, Luminal-type, and HER2-type) is scored according to their respective gene signatures (consisting of 28, 58, and 4 genes, respectively) reflecting specific functional pathways, with the highest score determining the subtype [1, 7]. In most cases, the highest score is significantly higher than the score of the other two subtypes, indicating a strong dominance of a single pathway activation in the tumor (so-called single subtype). However, in rare instances, the difference between the highest score and the second-highest score is statistically indiscernible, indicating that these tumors might be characterized by multiple activated pathways (dual subtype). Having a deeper understanding of which pathways are activated may help understanding the specific biology of BP dual subtypes that distinguish them from the single subtypes.

In addition to the standard BP subtypes (Basal-type, Luminal-type, and HER2-type), other studies have identified expression-based subtypes, which include normal-like, claudin-low, triple positive, and triple-negative [3, 8–11] types. Among others, Burstein and colleagues further classified the clinical triple-negative breast cancer (TNBC) subtype into basal-like immuno-activated (BLIA), basal-like immuno-suppressed (BLIS), luminal androgen receptor (LAR), and mesenchymal-like (MES) [12]. Therefore, assessing further the differences between the BP scores may help identifying additional subtypes previously not detected by standard BP. Also, understanding the biological characteristics of BP dual subtypes may help in guiding more effective treatment plans.

## Materials and methods

### Data

For this study, only data and no samples were collected, and all patient data were fully anonymized according to the ‘General Data Protection Regulation’ (GDPR) and the ‘Health Insurance Portability and Accountability Act’ (HIPAA) and are in compliance with the ‘Data Protection Act.’ This study was a retrospective analysis of (internal) studies between 2015 and 2020. These studies included those previously described in Beumer et al. [13], the FLEX registry trial (NCT03053193), the Neoadjuvant Breast Registry Symphony Trial (NBRST) (NCT01479101), and the Multi-Institutional Neo-adjuvant Therapy MammaPrint Project I (MINT) trial (NCT01501487). Most samples comply with MammaPrint (MP) eligibility criteria [14, 15], stage I, II, or operable stage III breast cancer, tumor diameter ≤ 5 cm , and up to three positive lymph nodes, with any ER/PR/HER2 status. Microarray processing was performed following standard procedure at Agendia [7] (Supplementary methods). Agendia’s customized diagnostic arrays were either a targeted array or a full genome array, as previously described [7, 13, 16].

Of the 15,580 samples analyzed with BP, 7985 had full-genome expression data available of which 1978 with clinicopathological information (Table 1). All samples analyzed with the targeted array had clinicopathological information available (Table 1b).

**Table 1:** (a) The BluePrint single and dual classification of the samples with full-genome data, which were used in differential expression analysis and their standard BluePrint classification (*n* = 7985) and (b) the BluePrint single and dual classification of the samples for which both the clinical information and the standard BluePrint classification are available

	Full genome	Standard BluePrint			Total
		Basal	Luminal	HER2	
*(a) Single–dual subtype classification*					
Basal-single	712	712	0	0	712
Luminal-single	6732	0	6732	0	6732
HER2-single	277	0	0	277	277
Luminal-Basal	122	51	71	0	122
Luminal-HER2	99	0	50	49	99
HER2-Basal	23	7	0	16	23
Luminal-HER2-Basal	20	5	11	4	20
Total	**7985**	775	6864	346	7985

	Full genome	Targeted array	Standard BluePrint			Total
			Basal	Luminal	HER2	
*(b) Single–dual subtype classification*						
Basal-single	150	440	590	0	0	590
Luminal-single	1727	6781	0	8508	0	8508
HER2-single	47	145	0	0	192	192
Luminal-Basal	32	124	58	98	0	156
Luminal-HER2	11	65	0	45	31	76
HER2-Basal	5	16	6	0	15	21
Luminal-HER2-Basal	6	24	10	13	7	30
Total	1978	**7595**	664	8664	245	9573

Of the 9573 samples, 1978 were processed on full genome and 7595 on targeted arrays. The total number of unique samples with BP classification is 15,580 (= 7985 + 7595) (highlighted in bold in the table) of which 15,087 are single subtypes, 449 are dual subtypes, and 44 are triple subtypes

The Neoadjuvant Breast Registry Symphony Trial (NBRST) [17–19] classified BC patients according to MP and BP and compared it with conventional IHC/FISH subtyping to predict treatment sensitivity. From the entire NBRST trial dataset (*n* = 1060), a subset that received HER2-targeted therapy (*n* = 289) was used to evaluate the association between the dual subtypes and response to HER2-targeted therapy. The NBRST trial protocol was approved by Institutional Review Boards at all participating sites (ClinicalTrials.gov NCT01479101). All patients consented to participation in the study and clinical data collection. Part of the anonymized data (BP results and IHC) used in this study was generated from early-stage BC patients collected from standard diagnostic testing and was only used to identify potential dual subtypes and not for any gene expression analysis. The data from studies can be shared by the authors upon reasonable request.

### BluePrint single and dual-subtype classification

Standard BP scores of 15,580 samples were calculated followed by dual-subtype classification, which was based on bootstrap technique [20], and multi-modality detection. Details on the procedure can be found in the Supplementary methods and Figure S1 (Fig. S1a).

### Conventional subtype classification

Clinicopathological information was available for 9573 of 15,580 samples, including IHC HR status for ER and PR, Ki-67, and IHC/FISH HER2 status (Table S1). Tumors with at least 1% positivity for either ER or PR were classified HR-positive (HR+), otherwise HR-negative (HR−). Tumors with HER2 IHC 0, 1+ or 2+ (FISH non-amplified) score were considered HER2-negative (HER2−) while tumors with HER2 IHC 2+ (FISH amplified) and 3+ score were considered HER2-positive (HER2+).

### Burstein classification

An algorithm published by Burstein et al., stratifies TNBCs into different subtypes by gene expression analyses of 80 signature genes. This algorithm was used to classify the Basal-single-type and Luminal-Basal-type samples into BLIA, BLIS, LAR, and MES [12].

### Software and statistics

Gene expression analysis was performed on full-genome microarray data (*n* = 7985) using limma (v3.2) [21]. Hallmark and Oncogenic gene sets from the Molecular Signatures Database v7.2 were used for gene set enrichment analysis (GSEA) [22]. Genes were ranked based on the effect size ratio using the Cohen’s *D* effect size [23]. Differentially expressed genes (DEG) were considered significant with a *p* value ≤ 0.05 and a log_2_ fold change ≥ 1.

Computational analysis and visualization were performed using R (v3.6.1) [24]. Principal component analysis (PCA) was performed using the “prcomp” package [25] (v3.6.2) and visualized using “ggplot” (v3.3.2) [26]. Unpaired, two-sample t-tests were used to measure if the means of ER, PR, and Ki-67 positivity were significantly different between single and dual subtypes. Chi-square test of Independence was used to test for differences of categorical variables within the Burstein classification (BLIA, BLIS, LAR, and MES) and a multivariate logistic regression analysis for response to therapy (pathological complete response, pCR) between single and dual subtypes. Molecular subtype classification algorithms were used from the “Genefu” package [27].

## Results

### BluePrint single and dual subtype classification

Molecular subtyping of patient tumors (*n* = 15,580) was performed at Agendia using the BP 80-gene assay as previously described [1, 7]. We applied the dual subtype classification method (see “Methods” for details) to assess the presence of multiple activated pathways. Most tumors were classified as single subtype (*n* = 15,087, 96.8%), followed by 449 (2.9%) tumors classified as dual subtype, and 44 (0.3%) tumors as triple subtype (Table 1). The most common dual subtypes in this dataset were the Luminal-Basal-type and the Luminal-HER2-type. These had sufficient numbers for downstream analyses while HER2-Basal-type and Luminal-HER2-Basal-type were not sufficient in size [28] and not further analyzed (Table 1a).

To note, only 1.9% of Luminal-type tumors were identified as dual subtype, whereas this was the case for 9.6% of the Basal-type and 23.8% of the HER2− type tumors. Since our dataset was largely HR+ HER2− (Table S1), in order to estimate the dual subtype prevalence in the overall BC clinical population, we iteratively created subsets representing expected distributions of clinical subtypes (https://seer.cancer.gov/statfacts/html/breast-subtypes.html) [29] (70% HR+ HER2−, 13% HR+ HER2+, 5% HR−/HER2+ and 12% HR-HER2−) and we detected 4.92% dual subtypes (95% CI 4.91–4.93) (Fig. S2).

### Principal component analyses using BluePrint reveals similarities between subtypes

**Fig. 1:**
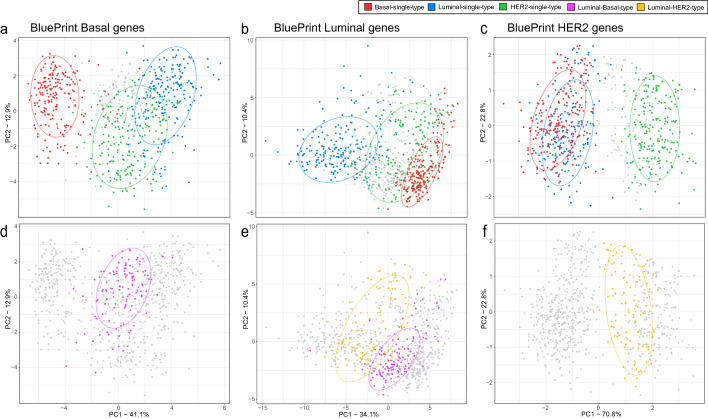
Principle component analysis using the three BluePrint signature gene sets (Basal-type, *N* = 28, panels **a** and **d**; Luminal-type, *N* = 58, panels **b** and **e**; HER2-type, *N* = 4, panels **c** and **f**). The *x*-axis shows variance explained for the first principle component (PC) and the *y*-axis show the variance explained for the second PC of the correspondent BluePrint signature gene set. **a**–**c** Clustering of Basal-single-type, Luminal-single-type, and HER2-single-type samples based on BluePrint signature genes. **d**–**f** Clustering of Luminal-Basal-type and Luminal-HER2-type based on BluePrint signature genes. **a**–**c** shows coloring of single subtype samples (blue, Luminal-single-type; green, HER2-single-type; red, Basal-single-type) whereas the dual subtype samples are colored grey. **d**–**f** shows this in reverse where the dual subtypes are colored (yellow, Luminal-HER2-type; pink, Luminal-Basal-type) and the single subtypes are shown in grey. The ellipses reported in each subfigure illustrate the 80% confidence intervals of the single and dual subtypes

To understand the similarities between single and dual subtypes, we performed PCA based on the BP gene expression signatures (Fig. 1a–e). We observed a clear distinction of single subtypes shown in the first two principal components (Fig. 1a–c). Luminal-Basal-type cluster separately from both Basal-single-type (Fig. 1d) and Luminal-single-type (Fig. 1e), conversely, Luminal-HER2-type (Fig. 1e, f) are more closely related with both Luminal-single-type (Fig. 1b) and HER2-single type (Fig. 1c).

### Differential gene expression analysis highlights differences between BluePrint dual and single subtypes

**Fig. 2:**
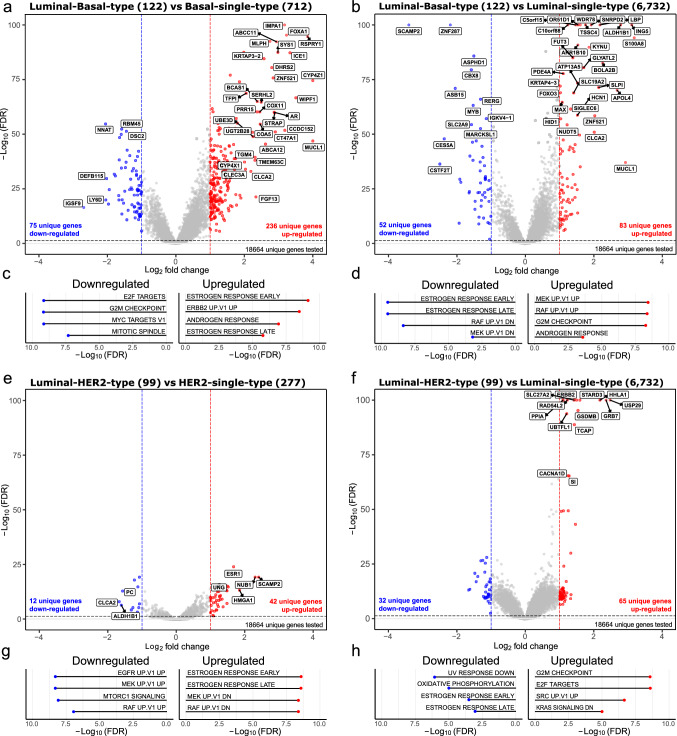
Differential gene expression analysis between BluePrint single and dual subtypes. The *x*-axis and *y*-axis report the Log_2_ fold change and the FDR-adjusted *p*-values (− Log_10_(FDR)), respectively. Number of tumor samples used for the analysis are shown in between brackets in titles. Significance thresholds of ≤ 0.05 FDR and a log_2_ fold change of ≥ 1 were used. Red and blue dots illustrate significant differentially expressed genes. The strongest differentially expressed genes are labeled (abs(logFC) ≥ 2 or −Log10 adj *p*-value ≥ 50). Differentially expressed genes are identified in the following comparisons: **a** Luminal-Basal-type versus Basal-single-type. **b** Luminal-Basal-type versus Luminal-single-type, **e** Luminal-HER2-type versus HER2-single-type, and **f** Luminal-HER2-type versus Luminal-single-type. Similarly, differentially expressed pathways are shown between **c** Luminal-Basal-type versus Basal-single-type. **d** Luminal-Basal-type versus Luminal-single-type, **g** Luminal-HER2-type versus HER2-single-type, and **h** Luminal-HER2-type versus Luminal-single-type. FDR = false discovery rate, UP = upregulated, DN = downregulated

Differential expression analysis using full-genome data (*n* = 7985) was performed to evaluate global transcriptional differences between single and dual subtypes. As expected from the PCA, when compared with their corresponding single subtypes, more DEGs were found for the Luminal-Basal-type (446 DEGs) (Fig. 2a, b) than for the Luminal-HER2-type (151 DEGs) (Fig. 2e, f).

Among the up-regulated genes in Luminal-Basal-type (vs. both Basal-single-type and Luminal-single-type) were present *MUCL1*, a known tumor suppressor gene [30], and *CLCA2,* a negative regulator of cancer cell migration and invasion [31].

Among the most up-regulated genes in Luminal-HER2-type compared with Luminal-single-type tumors, we found *GRB7*, *TCAP*, and *ERBB2* which belong to the HER2 amplicon and are known to be overexpressed in pathologically confirmed HER2 tumors [32]. Indeed, these genes were also up-regulated in HER2-single-type tumors (Fig. 2e). When comparing Luminal-HER2-type with HER2-single-type, *ESR1* was found to be upregulated, similarly as in Luminal-single-type tumors. Additionally, Luminal-HER2-type tumors were mainly classified as either Luminal B (*n* = 34/99, 34%) or HER2 enriched (*n* = 44/99, 44%) using the intrinsic subtype classifier of the”Genefu” [27, 33]. Together, these data suggest that both ER and HER2 are activated in Luminal-HER2-type tumors.

### Differences between BluePrint single and dual subtypes may impact therapy response pathways

A better understanding of the underlying biological characteristics of the dual subtypes may come from analyzing gene pathway regulation.

Comparison of Luminal-Basal-type with Basal-single-type revealed upregulation of two estrogen response (ESR) and one androgen response (AR)-related gene sets (Fig. 2c). Same ESR gene sets were downregulated in Luminal-Basal-type versus Luminal-single-type, indicating that Luminal-Basal-type has intermediate ER levels. Conversely, AR was upregulated in Luminal-Basal-type, versus both the Basal-single-type and Luminal-single-type. G2M and E2F pathways [34, 35] were either downregulated or upregulated in Luminal-Basal-type compared with Basal-single-type and Luminal-single-type, respectively, indicating that Luminal-Basal-type are less proliferative than Basal-single-type, but more proliferative than Luminal-single-type tumors. Taken together, Luminal-Basal-type tumors show a distinct biology from their single counterparts with decreased proliferation than Basal-single-type and AR activation.

Compared with single HER2-single-type tumors, a Luminal-HER2 type shows downregulation of MAPK (MEK and RAF) signaling pathways and ER activation (Fig. 2g). Clinical characteristics of the single and dual BP subtypes and their response to therapy may confirm these hypotheses and provide additional insights.

### BluePrint dual subtypes present clear clinicopathological differences from single subtypes

**Fig. 3:**
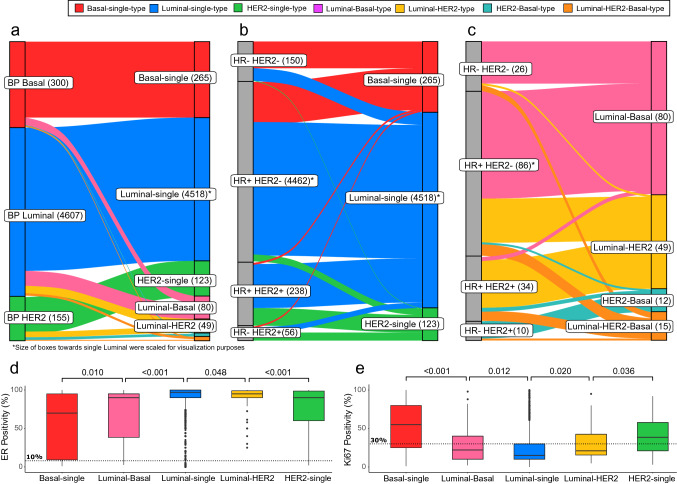
**a** Sankey plot showing the further stratification of the standard BluePrint (BP) Basal, Luminal, and HER2 subtypes with full-genome microarray data available, into the BP single and dual subtypes. **b** Sankey plot illustrating the re-classification of clinical-based subtypes (based on hormone receptors (HR) and human epidermal growth factor receptor 2 (HER2) status) to BP-based single-type molecular subtypes (Basal-single-type, Luminal-single-type, HER2-single-type). **c** Further stratification of the same clinical-based subtypes as in (**b**) to the BP-based dual subtypes (Luminal-HER2-type, Luminal-Basal-type, HER2-Basal-type, and Luminal-HER2-Basal-type). **d**, **e** Boxplots reporting for each single and dual subtype category (*x*-axis), the level and spread of estrogen receptor and Ki67 positivity based on Immunohistochemistry assessment (*y*-axis). Significant differential positivity between ER and Ki67 was assumed at a *p*-value < 0.05 determined with a t-test between subtype categories. To note, for 4511 of the 9573 tumor samples with clinical annotation, HR and HER2 status were not available (Table S1)

Standard BP Luminal-, HER2−, and Basal-type tumors were further stratified using the single–dual subtyping classification (Fig. 3a). Additionally, conventional clinical subtypes (based on IHC HR staining (ER, PR) and HER2 status) were further classified into BP single subtypes or dual subtypes (Fig. 3b, c).

Majority of HR-HER2− tumors were classified as Basal-single-type (*n* = 150/176) (Fig. 3b), while only 26 were dual subtype of which 22 were Luminal-Basal type.

Most of the HR+ HER2− tumors were classified as Luminal-single-type (*n* = 4285/4548), but interestingly, 3% (*n* = 152/4548) was classified as Basal-single-type, which corresponds to more than half of all Basal-single-types identified by BP (*n* = 152/265) (Fig. 3b). Of the HR+ HER2− with a dual subtype, majority was Luminal-Basal-type (*n* = 57/86) (Fig. 3c).

Most HR+ HER2+ tumors were classified as either Luminal-single-type (*n* = 165/272) or as HER2-single-type (*n* = 61/272) (Fig. 3b) with the most frequent dual subtype being the Luminal-HER2-type (*n* = 30/34) (Fig. 3c).

Luminal-single-type tumors had the highest IHC ER expression levels with the lowest levels observed in Basal-single-type tumors (Fig. 3d). Dual subtypes showed intermediate ER expression, compared to their single counterparts (Fig. 3d). ER low positive tumors (1–10% IHC) were mostly found in the Basal-single-type (*n* = 62/147, 42%) and in the Luminal-single-type (*n* = 58/147, 39%) (Fig. 3d). However, considering the differences in sample size of the subtypes, a larger fraction of Basal-single-type (24%) was found to be ER low positive, compared with other subtypes. Proliferation measured by % Ki-67 positivity was significantly higher in Luminal-Basal-type and Luminal-HER2-type compared with Luminal-single-type, but significantly lower than Basal-single-type and HER2-single-type (Fig. 3e). Indeed, there were significantly more Luminal-Basal-type (*n* = 34/47, 72.3%; *p* value < 0.001) than Basal-single-type tumors (59/173, 34.1%) with Ki67 < 30%, threshold recently proposed for the so-called TNBC low proliferation (TNLP) tumors [36] (Fig. 3e).

### Burstein LAR and MES subtypes are identified using BluePrint dual subtype classification

**Fig. 4:**
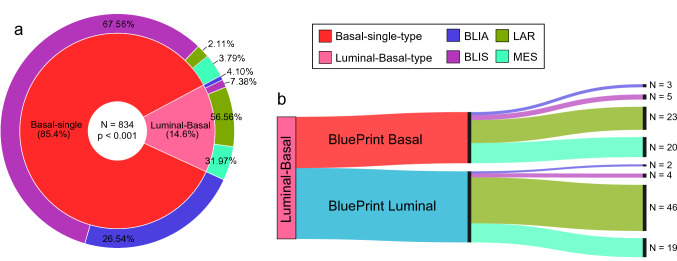
BluePrint (BP) dual subtype classification compared with Burstein’s classification of triple-negative breast cancer tumors [12]. **a** The inner circle contains percentages of the BP Basal-single-type and BP Luminal-Basal-type. The outer circle illustrates the correspondent Burstein classification into Basal-like immuno-activated (BLIA), Basal-like immuno-suppressed (BLIS), Luminal androgen receptor (LAR), or Mesenchymal (MES). **b**) Samples with the Luminal-Basal-type were split based on standard BluePrint classification to illustrate their distribution over BLIA, BLIS, LAR, and MES subtypes. Significant differential classification of Burstein subtypes was assumed at a *p*-value ≤ 0.05 determined with a Chi-Square test of Independence between subtypes

Since Luminal-Basal-type displays different transcriptional characteristics than Luminal-single-type and Basal-single-type, we classified them using the Burstein classifier to better understand their biology. Indeed, we found a significant association between BP single/dual subtypes and the Burstein BLIA, BLIS, LAR, and MES subtypes [12] (*p* value < 0.001) with the Basal-single-type classified mostly as BLIA or BLIS, whereas the Luminal-Basal-type as LAR or MES (Fig. 4a), irrespective of their standard BP subtype (Fig. 4b).

When using the PAM50 [3, 33] intrinsic subtype classifier of the”Genefu” [27] package, the Luminal-Basal-type tumors were mostly classified as HER2 enriched (HER2-e) (Table S3a). When comparing "Luminal-Basal/HER2-e” against “Luminal-Basal/ non-HER2-e”, common biomarkers for HER2 molecular classification were not differentially expressed (Table S3b). Indeed, ~ 98% of Luminal-Basal-type tumors were clinically HER2− (Fig. 3c).

### BluePrint dual subtype classification of the NBRST dataset shows refined prediction to therapy

**Fig. 5:**
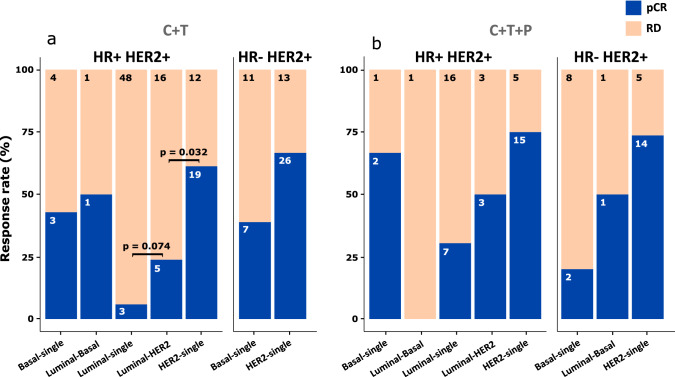
Distribution of pathologically confirmed HER2+ patients of the NBRST trial [18–20] based on the BluePrint single and dual subtype classification and their treatment response (*N* = 253). Patients are grouped based on their therapy regimen [chemotherapy (C) plus Trastuzumab (T) (panel **a**) or C + T and Pertuzumab (P) (panel **b**)], and their HR and HER2 status (HR+ HER2 + or HR- HER2+. The colored bars represents if a tumor did (pCR, blue) or did not [Residual Disease (RD), bisque] achieve pathological complete response (pCR). *p*-value determined with a chi-square test of independence between subtypes. Of the entire NBRST set (*n* = 289), 253 samples are showed due to low numerosity of HER2-Basal-type (*n* = 19) and Luminal-HER2-Basal (*n* = 17)

Our findings indicate that the Luminal-HER2-type shares clinical and genomic features with Luminal-single-type and HER2-single-type and previous studies suggest that HR and HER2 co-expression is associated with endocrine and HER2-targeted therapy resistance [37, 38]. Therefore, to better understand how Luminal-HER2-type relates to HER2-targeted therapy response, we analyzed the NBRST dataset (see Methods for details) [18] and selected only pathologically confirmed HER2+ tumors (*n* = 289) with gene expression and HER2-targeted therapy response data available [either Trastuzumab (T) only or with Pertuzumab (P)]. Patient tumors were stratified using the BluePrint dual subtype classification (Fig. 5).

BP HER2-single-type showed higher pCR rate to chemotherapy (C) + T compared to Luminal-HER2-type (61.3% vs. 23.8%, *p* = 0.032) (Fig. 5a). Although not significant (also due to lower numerosity of Luminal-HER2-type), this trend remained for patients that received additional P (Fig. 5b). Response rates of Luminal-HER2-type tumors was higher, but not significantly different than for Luminal-single-type. Instead, a significant higher response rate was observed for the HER2-single-type compared to the Luminal-HER2-type, after correcting for HR status, tumor stage, tumor grade, and therapy in a multivariate logistic regression analysis (*p* value = 0.006, Table S2).

## Discussion

Molecular subtyping using the standard BP 80-gene assay enables to discern the tumor subtype by the underlying functional pathways and not merely by HR and HER2 status [1, 7]. In most cases, the assay identifies a single, dominant activated pathway distinctive of a Luminal-, Basal-, or HER2-type tumor. This information often confirms the pathologically defined subtype but in many cases further classifies tumors from their initial clinical subtype into a different molecular subtype. This phenomenon has clinical implications for the treatment of patients, perhaps most notably in the ER+/Basal and HER2+/Luminal subtypes which have been previously described [18, 19, 39, 40].

The vast majority of the breast cancer tumors analyzed in this study using the BP test show a single activated pathway (i.e., single BP subtype) (97%); however, less frequently, they exhibit multiple activated pathways (i.e., dual or triple subtype) (3%), as we showed in a preliminary analysis [41]. Notably, this dataset mostly reflects a HR + population but upon sampling the data based on observed frequencies of clinical subtypes, such a percentage raises to ~ 5%. Importantly, the single and dual assessment performed on the NBRST dataset and also reported for the TRAIN2 [42] and APHINITY [43] patient cohorts show a higher number of dual subtypes, ranging from 11 to 30%, indicating that the dual subtype classification might have a greater clinical impact on a HER2+ population and that the potential clinical utility should be found in specific subgroups rather than in the entire EBC population. The analysis on the NBRST dataset was performed on limited numbers of dual subtypes (*n* = 32); however, the size was sufficient to generate statistically powerful results.

Overall, in this manuscript, we aimed to provide a better understanding of the biological diversity of EBC and these results should be taken with caution with respect to any immediate change in clinical management.

Next, by analyzing whole-transcriptomic data, we set out to understand if and how dual subtypes were distinct from single subtypes. For the analysis, we focused on the Luminal-Basal-type and Luminal-HER2-type tumors as the other dual subtypes were limited in size.

Neither the Basal nor the Luminal BP template genes were able to fully capture the biology of the Luminal-Basal-type tumors. The majority of tumors expressing typical Basal gene patterns are TNBC by pathology [44], and it is known that there is a large overlap between BP Basal subtypes and TNBCs. Therefore, we applied the TNBC Burstein classifier on the Basal-single-type and Luminal-Basal-type. Basal-single-type tumors were mostly classified as BLIA and BLIS while Luminal-Basal-type tumors were more likely to be either LAR or MES. Genes described by Burstein et al. to be up-regulated in the LAR subtype, such as *DHRS2*, *AGR2, FOXA1*, *AR,* and *MUCL1,* were indeed higher expressed in Luminal-Basal-type compared with the Basal-single-type samples. Since the majority of Luminal-Basal-type tumors were classified as LAR, and according to Burstein et al., those patients derive benefit from traditional anti-estrogen or anti-androgen therapy, we could speculate that Luminal-Basal-type cancers would benefit from such treatment as well. Furthermore, *ADH1B* and *FABP4* genes were up-regulated in Luminal-Basal-type samples compared with Basal-single-type samples. The upregulation of these genes is typical of the MES subtype, which is characterized by the dysregulation of cell cycle and DNA damage repair pathways. On the contrary, BLIS subtype-specific genes, *HORMAD1*, *SOX10*, *SERPINB5,* and *FOXC1*, were up-regulated in Basal-single-type samples compared with Luminal-Basal-type samples. Therefore, we could hypothesize that among the Basal-single-type samples, two subgroups are present which are indiscernible with the current dual subtype classification, but might have a different prognosis according to Burstein et al. and require additional analyses. Notably, majority of the Luminal-Basal-type showed a Ki67 positivity below 30% which might indicate that they share features with the TNLP tumors recently described by Bhargava and colleagues [36]. Additionally, no large agreement was found between any of the dual subtypes and the normal-like [3, 4] (Table S3) or claudin-low classifications [9, 27] (data not shown). Conversely, BluePrint Basal-, Luminal-, and HER2-single type classifications were largely concordant with the intrinsic subtypes (> 90%) (see Table S3). Interestingly, and perhaps unexpectedly, the Luminal-Basal-type tumors were mostly classified as HER2-e intrinsic subtype, possibly due to the absence of Luminal- and Basal-type biology in the BP Luminal-Basal-type.

Luminal-HER2-type samples consistently showed patterns of both ER and HER2 activation (by expression and IHC/FISH), which may suggest similarities to the clinically triple-positive tumors [10]. Expression of both ER and HER2 may lead to receptor crosstalk which has often been associated with resistance to both endocrine and HER2-targeted therapies [45]. However, down-regulation of the MAPK-related gene sets MEK and RAF may indicate no downstream activation of the HER2 pathway. Therefore, Luminal-HER2-type tumors are unlikely fueled through the HER2 pathway alone and HER2-targeted therapies might not be as effective as in the HER2-single-type tumors. This suggestion is strengthened by the observation in the NBRST data that Luminal-HER2-type tumors have a significantly lower pCR rate to neoadjuvant chemotherapy including HER2-targeted agents compared with HER2-single-type tumors (p-value < 0.032). This is supported by preliminary subanalysis of the TRAIN2 [42, 46] and APHINITY [43, 47, 48] trial datasets, suggesting that BluePrint HER2-single-type tumors derive the most benefit from HER2 dual-targeted treatment [43].

It has been suggested that clinically triple-positive tumors develop endocrine resistance as downstream-activated MAPK inhibits ER transcription and phosphorylates ER [38]; however, in this study, Luminal-HER2-type tumors may be only driven by the ER pathway, as MAPK is downregulated compared with HER2-single-type tumors and not significantly different from that of Luminal-single-type tumors. Further analysis on Luminal-HER2-type samples treated with endocrine therapy is required to investigate and confirm this hypothesis.

## Conclusion

Our study showed that by further dissecting the BP scores, it is possible to identify a small proportion of EBCs that have dual-activated BP pathways. These dual subtypes display specific transcriptional and clinicopathological features supporting the idea that they represent a different biological subgroup than their single counterparts. Most dual BP subtypes are either Luminal-Basal-type or Luminal-HER2-type.

The Luminal-Basal-type shows lower proliferation levels compared with the Basal-single-type and AR activation. Interestingly, using the Burstein classification, Luminal-Basal tumors are mostly classified as LAR and MES subtypes.

The Luminal-HER2-type resembles features of both the Luminal-single-type and HER2-single-type. However, patients with Luminal-HER2-type tumors have a lower pCR rate after receiving HER2-targeted therapies in addition to chemotherapy compared with patients with a HER2-single-type.

Taken together, BP dual classification shows potential clinical utility in helping treatment decision for a limited, but still relevant, fraction of EBC patients with dual subtypes that may benefit from additional or alternative targeted therapies. Even though molecular subtyping is not yet standardly used in routine clinical diagnostics, increasing number of evidences are emerging indicating that molecular subtypes should become part of breast cancer management [49]. In this light, results presented here further support the need toward such transition and implementation.

Future work will be focused on further confirming and prospectively validating the findings described here in additional independent datasets.

## Supplementary Information

Below is the link to the electronic supplementary material.

Supplementary file1 (PDF 376 KB)

## Abbreviations

BP: BluePrint
ER: Estrogen receptor
PR: Progesterone receptor
HER2: Human epidermal growth factor receptor
IHC: Immunohistochemistry
FISH: Fluorescence in-situ hybridization
EBC: Early-stage breast cancer
MP: MammaPrint
PCA: Principal component analysis
DEG: Differentially expressed gene
TNBC: Triple-negative breast cancer
LAR: Luminal androgen receptor
MES: Mesenchymal
BLIA: Basal-like immuno-activated
BLIS: Basal-like immuno-suppressed
FDR: False discovery rate
GSEA: Gene set enrichment analysis
