# Supplementary material for: BluePrint breast cancer molecular subtyping recognizes single and dual subtype tumors with implications for therapeutic guidance
Source: Breast Cancer Res Treat. 2022 Aug 19;195(3):263–74. doi: 10.1007/s10549-022-06698-x (PMC9464757; doi:10.1007/s10549-022-06698-x)
Supplement: Supplementary file 1 — Supplementary file1 (PDF 376 KB) [file 10549_2022_6698_MOESM1_ESM.pdf]

BluePrint Breast Cancer Molecular Subtyping Recognizes Single and Dual Subtype Tumors with Implications for Therapeutic Guidance

Authors: Midas M Kuilman[¥1], Architha Ellappalayam[¥ 1], Andrei Barcaru[1], Josien C Haan[1], Rajith Bhaskaran[1], Diederik Wehkamp[1], Andrea R Menicucci[2], William M Audeh[2], Lorenza Mittempergher[1*] AND Annuska M Glas[1*]

Affiliations:

1. Department of Research & Development, Agendia N.V, Radarweg 60, 1043NT, Amsterdam, The Netherlands
2. Department of Medical Affairs, Agendia Inc, 22 Morgan, CA 92618 Irvine, USA

¥ Equal contribution as first authors
*Corresponding authors

Mittempergher ORCID ID: 0000-0003-3425-3965

Glas ORCID ID: 0000-0002-0775-138X

Corresponding authors:
Lorenza Mittempergher and Annuska M Glas
lorenza.mittempergher@agendia.com and annuska.glas@agendia.com
Tel: 31 20 462 1566 and +31 20 462 1520
Agendia NV, Radarweg 60, 1043 NT, The Netherlands

Supplementary Information

Supplementary methods

- **Microarray processing**

Microarray processing was performed following standard procedure at Agendia [7]. Briefly, Total RNA was isolated from Formalin-Fixed-Paraffin-Embedded (FFPE) tissue with the RNeasy FFPE kit (Qiagen), DNase treated and amplified using a TransPLEX C-WTA kit (Rubicon Genomics, Ann Arbor, MI). Amplified cDNA was labeled using the Genomic DNA Enzymatic Labeling Kit (Agilent Technologies, Santa Clara, CA) and hybridized onto Agendia's diagnostic arrays (custom-designed, Agilent Technologies), according to the manufacturer's instructions.

- **BluePrint single and dual-subtype classification**

Dual-subtype classification was performed as follows and it is visually summarized in Figure S1. First the standard BluePrint (BP) score was calculated from 15580 samples as previously described (Figure S1a-b) [7]. Briefly, for each tumor, three scores were generated, and the subtype with the highest score was the categorical subtype reported. Next, BP scores were scaled with a SoftMax function (*Goodfellow IJ, et al (2016) 6.2.2.3 SoftMax Units for Multipouli Output Distributions. Deep Learning. MIT Press. pp 180-184*) (Fig S1c) to reduce variance and outlier impact, which allows for optimal threshold determination between single and dual subtypes. Using a bootstrap algorithm [20]**,** samples were divided into 70% and 30% groups per BP subtype for 1000 iterations (FigS1d). For each iteration, the two highest scores were selected (Fig S1e) and the distance between them was calculated (Fig S1f). The distribution of the differences between BP scores was constructed (Fig S1g). If a bimodal distribution emerged (implying the presence of single and dual subtypes), the separation point (*i.e.,* local minimum) between the two distributions was selected as a threshold candidate (Fig S1h). After 1000 bootstrap iterations, multiple threshold candidates were captured for each subtype. The maximum likelihood values of threshold distributions were taken as thresholds for the identification of dual subtypes (Fig S1i), which are reported in FigS1j. If the difference between the two highest BP SoftMax scores was lower or equal to the corresponding single-dual threshold, then the tumor was classified as a dual subtype comprised of the two highest molecular subtype scores. If tumors had similar scores for all three subtypes, they would be defined as triple subtype.

Supplementary Tables and Figures

Table S1: Clinical-pathological characteristics of the patients analyzed in this study.

	Standard BluePrint classification			
	Luminal (n=8664)	HER2 (n=245)	Basal (n=664)	All (n=9573)
**Age at diagnosis (years, median, range)**	62 (23-93)	61 (23-95)	58.5 (23-87)	62 (23-95)
**Nodal Status**				
0	1516	21	121	1658
1	1320	24	47	1391
2	335	2	6	343
3+	163	4	7	174
Missing	5330	194	483	6007
**Grade**				
1	1409	6	8	1423
2	2966	58	74	3098
3	869	66	309	1244
Missing	3420	155	273	3848
**Clinical subtype based on receptor status**				
HR+ HER2-	4343	33	172	4548
HR+ HER2+	180	83	9	272
HR- HER2+	20	37	9	66
HR- HER2-	64	2	110	176
missing	4057	90	364	4511
**Ki67 Percentage**				
Median positivity (1st - 3rd quantiles)	16 (10-30)	40 (21-60)	50 (20-80)	18 (10-32)

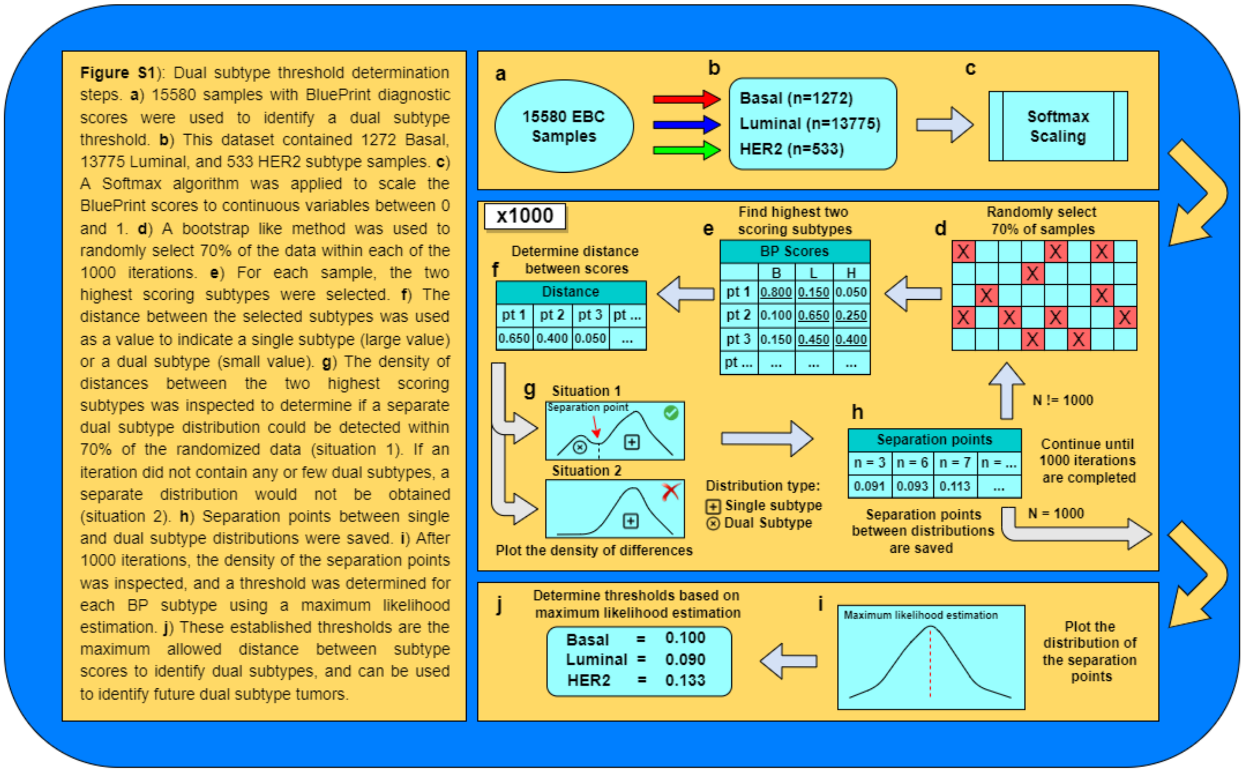

Figure S1): Dual subtype threshold determination steps. **a**) 15580 samples with BluePrint diagnostic scores were used to identify a dual subtype threshold. **b**) This dataset contained 1272 Basal, 13775 Luminal, and 533 HER2 subtype samples. **c**) A Softmax algorithm was applied to scale the BluePrint scores to continuous variables between 0 and 1. **d**) A bootstrap like method was used to randomly select 70% of the data within each of the 1000 iterations. **e**) For each sample, the two highest scoring subtypes were selected. **f**) The distance between the selected subtypes was used as a value to indicate a single subtype (large value) or a dual subtype (small value). **g**) The density of distances between the two highest scoring subtypes was inspected to determine if a separate dual subtype distribution could be detected within 70% of the randomized data (situation 1). If an iteration did not contain any or few dual subtypes, a separate distribution would not be obtained (situation 2). **h**) Separation points between single and dual subtype distributions were saved. **i**) After 1000 iterations, the density of the separation points was inspected, and a threshold was determined for each BP subtype using a maximum likelihood estimation. **j**) These established thresholds are the maximum allowed distance between subtype scores to identify dual subtypes, and can be used to identify future dual subtype tumors.

Table S2: Multivariate logistic regression analysis to examine the subtype, HR status, tumor grade, tumor stage, and treatment variables to determine those that best predict response to HER2-targered therapy. Only cases with complete clinical information were used.

			Exp (B)	95% C.I. for EXP (B)		Significance	N
				Lower	Upper		
Variables		Intercept	5.698	1.454	26.466	0.018	
	**Subtype**	Single HER2*	-	-	-	-	101
		Luminal-HER2	0.204	0.062	0.619	0.006	25
	**HR status (IHC)**	ER negative*	-	-	-	-	56
		ER Positive	0.571	0.215	1.466	0.249	70
	**Tumor grade**	Grade II*	-	-	-	-	51
		Grade III	0.982	0.422	2.251	0.967	75
	**Tumor stage**	Stage I*	-	-	-	-	21
		Stage II	0.629	0.173	2.020	0.453	70
		Stage III	0.154	0.033	0.634	0.013	24
		Stage IV	0.295	0.052	1.658	0.160	11
	**Treatment**	C + T*	-	-	-	-	83
		C + T + P	1.844	0.768	4.632	0.179	43

* Used as reference in the odds ratio calculation.

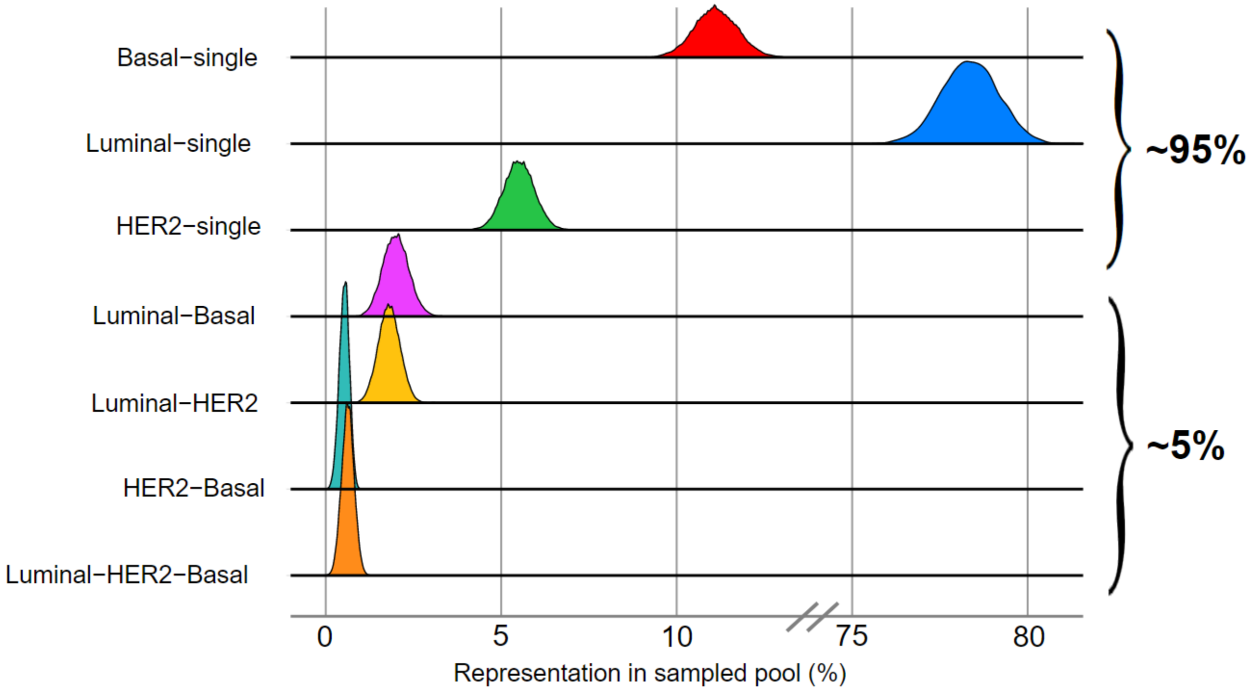

Figure S2: Distribution of the representation of single and dual subtypes in a sampled pool of tumor samples. The x-axis report the proportion in percentage for each subtype (y-axis). Curly brackets on the right indicate the subtype prevalence obtained using the sampled pool. .Sampled pool represents actual occurrences of clinical subtypes (70% HR+HER2-, 13% HR+HER2+, 5% HR-/HER2+ and 12% HR-HER2-) according to literature (https://seer.cancer.gov/statfacts/html/breast-subtypes.html) [29].

Table S3a: Distribution of dual subtypes over molecular subtypes of Genefu.

		Genefu molecular subtyping classification				
		Luminal A	Luminal B	HER2-e	Basal	Normal-like
Single-dual subtype classification	Basal-single-type	0	2	42	660	8
	Luminal-single-type	3722	2303	163	109	435
	HER2-single-type	6	21	243	6	1
	Luminal-Basal-type	11	14	63	12	22
	Luminal-HER2-type	16	34	44	3	2
	HER2-Basal-type	0	0	14	8	1
	Luminal-HER2-Basal-type	0	13	13	3	1

Table S3b: Log_2 fold change and adjusted P value of 4 HER2 related genes by comparing Genefu HER2-e BP Luminal-Basal against Genefu non- HER2-e BP Luminal-Basal tumors.

Gene	Log fold change	Adjusted P value

ERBB2	0.346	0.138
GRB7	0.230	0.199
TCAP	-0.049	0.640
STARD3	0.199	0.159
